# Refractive Index Sensing Using Helical Broken-Circular-Symmetry Core Microstructured Optical Fiber

**DOI:** 10.3390/s22239523

**Published:** 2022-12-06

**Authors:** Mingjie Cui, Zhuo Wang, Changyuan Yu

**Affiliations:** 1 Department of Electronic and Information Engineering, Photonics Research Institute, The Hong Kong Polytechnic University, Kowloon, Hong Kong; 2 Department of Electrical Engineering, Photonics Research Institute, The Hong Kong Polytechnic University, Kowloon, Hong Kong; 3 The Hong Kong Polytechnic University Shenzhen Research Institute, Shenzhen 518057, China

**Keywords:** microstructured optical fiber, helical core fiber, fiber sensing

## Abstract

Helical twist provides an additional degree of freedom for controlling light in optical waveguides, expanding their applications in sensing. In this paper, we propose a helical broken-circular-symmetry core microstructured optical fiber for refractive index sensing. The proposed fiber consists of pure silica and its noncircular helical core is formed by a broken air ring. By using finite element modeling combined with transformation optics, the modal characteristics of the fiber are investigated in detail. The results show that for the core located at the fiber center, the confinement loss of fundamental core modes increases with twist rate, whereas for a sufficiently large core offset the modes can be well confined owing to the twist-induced light guidance mechanism, showing decreases with rising twist rate in the loss spectra. Moreover, we have found that for large twist rates and core offsets, resonant peaks occur at different twist rates due to the couplings between the fundamental core modes and the highly leaky modes created by the helical structure. The refractive index sensing performance is also studied and the obtained results show that the proposed fiber has great potential in fiber sensing.

## 1. Introduction

Twisting an optical fiber can offer new opportunities for light controlling and shaping. It is shown that circular birefringence and single mode transmission with a large normalized frequency can be achieved by using a twisted fiber with a helical off-center core, which was fabricated by rotating during the fiber drawing process, in 1987 [1]. Afterwards, studies on the couplings between core and cladding modes in helical fibers drew much attention. Based on the mode couplings, researchers found that the fibers can be used in various applications, such as polarizers, couplers, and sensors [2,3,4,5,6]. Further, studies were also conducted on the helical fibers with rotational symmetric noncircular fiber core owing to the rotational symmetry of the core, which can affect the couplings between core and cladding modes greatly. More recently, the fiber processing method developed along with the progress in microfabrication, a helically twisted photonic crystal fiber (PCF), has been reported by Shin et al. [7] and in 2012, a detailed interpretation from the terms of mode couplings was given by Wong et al. [8]. Later, in 2013, it was shown that the helically twisted PCFs can be used for twist and strain sensing [9]. In 2014, Xi et al. presented a helically twisted three-fold symmetry Y-shaped core PCF, showing that helical Bloch modes carrying orbital angular momentum (OAM) can be well-preserved in the fiber, and demonstrated the stable transmission of first-order OAM modes in experiments [10]. In 2016, it was shown that helically twisted PCFs also can serve as current sensors due to their circular birefringence [11]. Meanwhile, new guidance mechanism was discovered in a coreless photonic crystal fiber. It was demonstrated that the light can be robustly confined in such fibers without any cores and the guided modes can be controlled by changing the twist rate [12]. In 2019, light guiding in a microstructured optical fiber with a partially open ring of holes was reported by Napiorkowski et al., and the experimental study showed that the fiber could be used for displacement sensing [13]. To date, the potential of helically twisted PCFs has been broadened to many applications, such as circular polarizers [14], vortex generators [15,16] and fiber lasers [17,18]. Particularly, the expansion of sensing applications can benefit greatly from the studies on the light guidance mechanism in helical microstructured optical fibers [12,13,19].

In this paper, we propose a helical broken-circular-symmetry core microstructured optical fiber for refractive index sensing. It is made of pure silica with a compact structure, and a broken air ring forms an incomplete barriered core. The fiber is twisted along the propagation axis. Modal characteristics relative to the twist rate are investigated in detail. The numerical results show that for the broken air ring located in the fiber center, the fundamental core modes exhibit increasing overall confinement loss with twist rate since the helical structure cannot provide any additional confinement for the light guiding in this case. However, for the broken air ring placed a distance away from the fiber center, the confinement of the modes at the center of the ring will be enhanced due to the twist-induced light guidance mechanism, showing a decrease in the loss spectrum. Moreover, we first observe the couplings between the core modes and the leaky modes generated by the helical microstructure appeared at some twist rates. We have also studied the variations in the loss spectra by changing the refractive index of analyte filled in the air ring. The variation of modal loss shows a high linearity to the changing of refractive index of analyte and the maximum amplitude sensitivity shown in this paper can reach over 300 RIU^−1^. The obtained results indicate that the proposed fiber has great potential in sensing applications.

## 2. Modeling Methods

To study the modal characteristics of an optical fiber with periodically helical structure, it was a good choice to use the finite element method combined with the transformation optics formalism. As mentioned in [20], the calculation performed in a helical coordinate system rather than the Cartesian coordinate system would benefit us significantly. The relations between helicoidal coordinates (*ξ*_1_, *ξ*_2_, *ξ*_3_) and Cartesian coordinates (*x*, *y*, *z*) are given by the following equations:

(1) $$\left\{ \begin{array}{l} x=\xi_{1}\cos(\alpha z)+\xi_{2}\sin(\alpha z)\\ y=-\xi_{1}\sin(\alpha z)+\xi_{2}\cos(\alpha z)\\ z=\xi_{3} \end{array}\right.$$

where α = 2π/Λ is the twist rate and Λ denotes the helix pitch.

The materials that are isotropic and homogeneous in the Cartesian space can be transformed to the helicoidal coordinate system so that the form of Maxwell’s equation remains unchanged in the helical frame. The equivalent transformation of the material properties between the two coordinate systems is given by:

(2) $$\left\{ \begin{array}{l} \left[\varepsilon \right]=\varepsilon T^{-1}\\ \left[\mu \right]=\mu T^{-1} \end{array}\right.$$

where [*ε*] and [*μ*] represent the permittivity and the permeability in the helical coordinates, respectively, and the metric tensor *T* is given by:

(3) $$T=\left(\begin{array}{ccc} 1 & 0 & \alpha \xi_{2}\\ 0 & 1 & -\alpha \xi_{1}\\ \alpha \xi_{2} & -\alpha \xi_{1} & 1+\alpha^{2}(\xi_{1}^{2}+\xi_{2}^{2}) \end{array}\right)$$

It is evident that the equivalent permittivity [*ε*] and permeability [*μ*] in the helicoidal coordinates only depend on *ξ*_1_ and *ξ*_2_ in this case. Therefore, a three-dimensional model here can be represented by a two-dimensional one, which in fact simplifies the numerical calculations greatly without losing any information on the electromagnetic phenomena. Moreover, for a boundary condition surrounding the region of interest in the helical frame, a twisted perfectly matched layer is used to absorb the scattering light and estimate the confinement loss of modes in the fiber [21,22].

## 3. Fiber Structure

**Figure 1:**
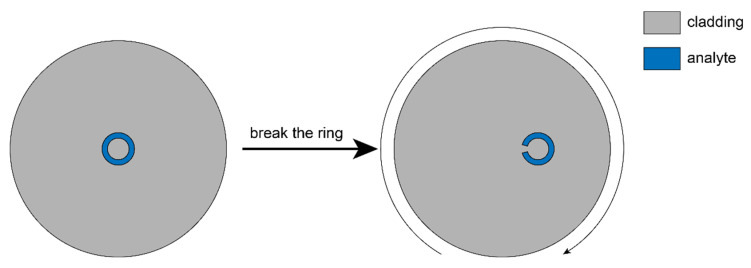
Cross-section of an imaginary fiber (**left**) and the proposed fiber (**right**). The curved arrow indicates the twist direction.

A cross-section of the proposed fiber is shown in Figure 1. It consists of a broken air ring that can be treated as a twelfth part of one complete air ring that is removed, forming a non-circular core without a complete refractive index barrier. The diameters of the inner circle and outer circle are R_1_ = 3 μm and R_2_, respectively. The broken air ring is also symmetric about the fiber axis of symmetry. Note that no bound mode can be well-confined in such a fiber core. Different core offsets are investigated in the following sections, and *ρ* denotes the distance between the fiber center and the broken air ring. The air ring is filled with analyte of refractive index *n_a_*, and it equals air when nothing is filled in the ring. The fiber is helically twisted along the axial direction of the *Z*-axis, and the cladding is made of pure silica. The helical fiber can be fabricated by spinning the preform during the drawing process. Here, the wavelength-dependent refractive index of fused silica is given by the Sellmeier equation:

(4) $$n(\lambda )=\sqrt{1+\sum_{i=1}^{3}\frac{A_{i}\lambda^{2}}{\lambda^{2}-Z_{i}^{2}}}$$

where *λ* is the wavelength in microns, *A_i_* and *Z_i_* are the coefficients whose value can be found in [23]. The operating wavelength of 1.55 μm is used by default. 

## 4. Results and Discussions

**Figure 2:**
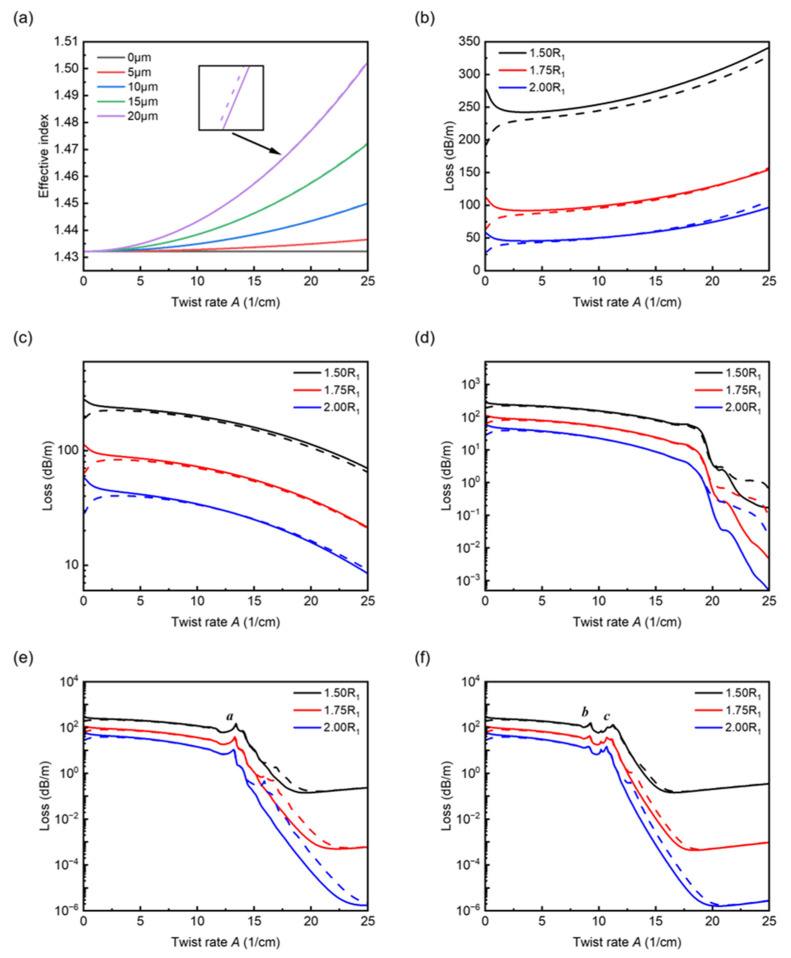
Effective indices and loss spectra of the $HE_{11}^{-}$ (solid lines) and $HE_{11}^{+}$ (dash lines) fundamental modes for five core offsets. (**a**) Effective indices versus twist rate with R_2_ = 1.5R_1_. (**b**–**f**) Loss spectra for core offsets *ρ* = 0, 5, 10, 15 and 20 μm with R_2_/R_1_ varying from 1.5 to 2.0.

First, we have calculated the effective indices and the loss spectra of the HE_11_ fundamental modes in the proposed fibers without any analyte filled in at five different core offsets, including the one located at the fiber center. Meanwhile, the ratio R_2_/R_1_ changes from 1.5 to 2.0. In Figure 2a, we present the effective index *n*_eff_’ versus the twist rate *A*, and the obvious changes in leakage loss with respect to the twist rate *A* are given in Figure 2b–g. Here, *A* is the twist rate *α* in a reduced form, expressed as *A* = α/(2π).

As shown in Figure 2a, the effective indices of fundamental modes increase sharply with twist rate, and for larger core offsets, the rises become more dramatic in the effective index, while for the ring located at the fiber center, the effective index basically stays the same. This is because the light is forced to travel a longer helical path when a normal fiber turns into a twisted one, leading to an increase in the effective index. Note that the effective indices presented in Figure 2a are with R_2_ = 1.5R_1_ since the change in the air ring ratio has a very minor effect on the effective indices. The effective index of core modes with sufficiently large core offsets in helical-core fibers can be approximated by the following equation [24]:

(5) $$n_{eff}\prime =n_{eff}\sqrt{1+\alpha^{2}\rho^{2}}+m\frac{\lambda }{2\pi }\alpha$$

where *ρ* is distance between the core and fiber center, *m* is the azimuthal order relative to the total angular momentum and, for fundamental core modes, takes the value ±1 representing two opposite handedness based on the circular polarization. One can see from Equation (5) that the elongation of the optical path mainly depends on the product factor *αρ*, and therefore, it is understandable for the broken air ring located at the fiber center (*ρ* = 0 μm) that the effective index remains unchanged. Figure 2b shows the variation in confinement loss with twist rate for *ρ* = 0 μm. It is quite clear that the overall loss increases with twist rate, though the core modes are already at a high leakage loss when the fiber untwisted. This is because the fiber twist enhances the mode leakage through the gap in the broken air ring. However, in the case of *ρ* = 5 μm, one can see that the loss decreases with twist rate, showing loss characteristics contrary to those in the case of *ρ* = 0 μm. For sufficiently large core offsets, mode deformations will arise owing to the fiber twist of the helical core fibers. Specifically, they mainly manifest themselves as the mode field moving outwards with respect to the fiber center. In the case of the proposed fiber here, the fiber twist reduces the leakage from the gap greatly, and the broken air ring serves as a refractive index barrier preventing the light from leaking out in other directions. Hence, the broken air ring plays a role in reducing the confinement loss in this case. The trend of curves presented in Figure 2d is basically the same as that in Figure 2c, apart from the slight distortions that appear in the twist rate range from 15 cm^−1^ to 20 cm^−1^. By analysis, together with Figure 2d–f, we have confirmed that the distortions/peaks that occurred in the loss spectra are due to the couplings between the core modes and the highly leaky mode created by the helical structure. With R_2_ increasing, one can see that the confinement loss decreases significantly but the trends of the curves are basically the same since the increase in the proportion of air mainly enhances the confinement of mode fields.

**Figure 3:**
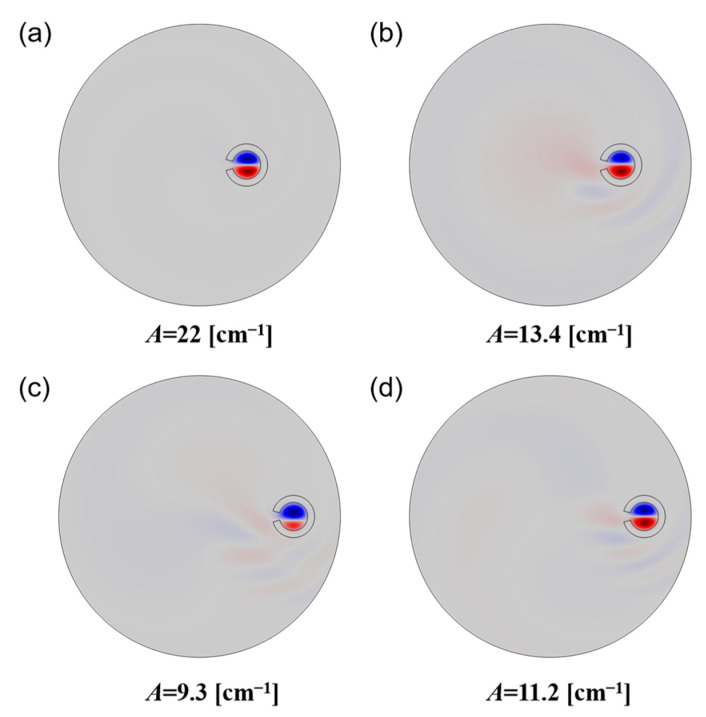
Transverse component of the electric field for chosen $HE_{11}^{-}$ core modes with R_2_ = 1.5R_1_ and *ρ* = 10 μm (**a**), *ρ* = 15 μm (**b**) and *ρ* = 20 μm (**c**,**d**).

As shown in Figure 3a, away from the loss peak, one can see that the light can be well-confined by the broken air ring with a large *αρ* factor. Figure 3b–d show the transverse electric field distributions corresponding to the significant loss peaks *a*, *b*, and *c* marked in Figure 2e,f. Compared to the field distribution shown in Figure 3a, one can clearly see the interactions between the core modes and the highly leaky modes outside. Moreover, with the increasing *αρ*, the resonant peaks tend to increase and shift to the shorter twist rate. Note that the two core modes with opposite handedness appear to have the same behaviors in the curves, indicating that the optical phenomena occurring here are basically polarization-insensitive. 

Next, we perform a simulation of the fibers with analyte filled in the broken air ring. The refractive index of analyte changes from 1.33 to 1.40. Here, as a contrast to Figure 2, we begin with showing the effective indices and loss spectra of the fundamental modes with core offset changing from 0 to 20 μm and R_2_/R_1_ varying from 1.5 to 2.0. 

**Figure 4:**
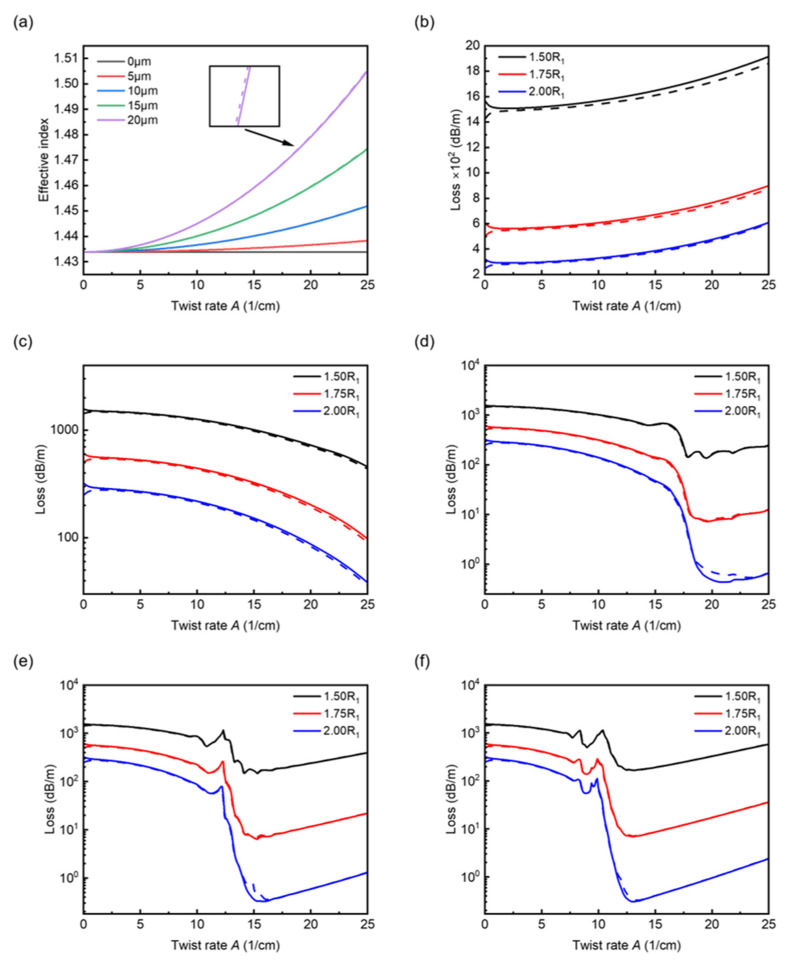
Effective indices and loss spectra of the $HE_{11}^{-}$ (solid lines) and $HE_{11}^{+}$ (dash lines) fundamental modes for *n_a_* = 1.33 with five core offsets. (**a**) Effective indices versus twist rate with R_2_ = 1.5R_1_. (**b**–**f**) Loss spectra for core offsets *ρ* = 0, 5, 10, 15 and 20 μm with R_2_/R_1_ varying from 1.5 to 2.0.

**Figure 5:**
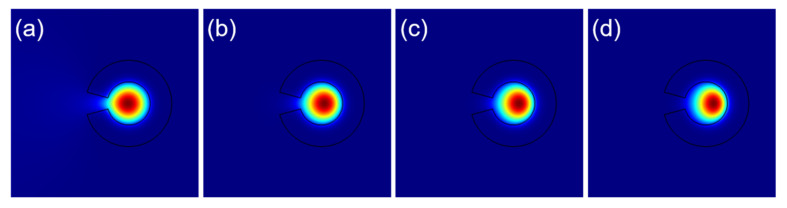
Mode profiles calculated for R_2_ = 2R_1_, *ρ* = 15 μm and twist rate *A* = 0 (**a**), 15 (**b**), 20 (**c**) and 25 cm^−1^ (**d**).

As shown in Figure 4a, the trends in effective indices are basically the same as that in Figure 2a, except the overall magnitude increased in this case due to the analyte of refractive index filled in the air ring. The overall confinement loss illustrated in Figure 4b–f is apparently much higher than that in Figure 2b–f. This is owing to the rise of refractive index in the air ring causing the broadening of the mode field, leading to the increase in loss spectra. The trends of curves in loss spectra also do not change too much compared to the curves in Figure 2b–f. However, for Figure 4e,f one can see that the loss gradually increases again at some twist rates under the coupling conditions. Specifically, in Figure 4e, for twist rate *A* larger than 15 cm^−1^ approximately, the curves begin to rise. The same case occurs in Figure 4f for twist rate *A* larger than 13 cm^−1^ approximately. The same trend also appears in Figure 2f, which is not as significant as that shown in Figure 4e,f. The increases in the loss spectra in these cases can be attributed to the modal displacement towards the outside, which means the modal fields tend to leak into the air ring. This can be regarded as a loss amplification effect due to the fiber twist. The mode profiles are given in Figure 5, demonstrating the evolution of mode profiles with changing twist rate.

**Figure 6:**
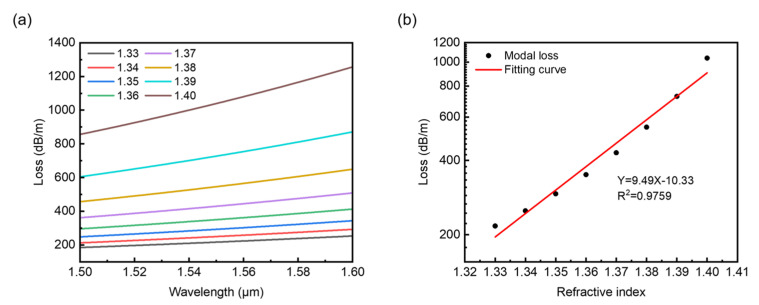
(**a**) Wavelength-dependent loss spectra with analyte refractive index changing from 1.33 to 1.40. (**b**) Linear fitting of loss increasing with refractive index *n*_a_ for wavelength 1.55 μm.

To study the effect of changes in the refractive index of analyte *n*_a_ on the core modes, we select the fiber structural parameters with R_2_ = 2R_1_, *ρ* = 15 μm for the following sections unless specified, and with *n*_a_ varying from 1.33 to 1.40 for some fixed twist rates. As shown in Figure 6a, for twist rate *A* = 5 cm^−1^, one can see that the losses gradually increase with wavelength due to the fiber twist. More importantly, for a fixed wavelength, the confinement loss increases significantly with *n*_a_ changing from 1.33 to 1.40 in the wavelength range from 1.5 to 1.6 μm, showing that the light confined by the ring is sensitive to the changes in refractive index of the surroundings. It is not difficult to find that the increases in the loss spectra are nonlinear with *n*_a_ changing from 1.33 to 1.40. However, we have found that the loss spectra might show good linearity in the log scale. The linearity and a sensitivity of curve slope correlation for selected wavelength 1.55 μm are shown in Figure 6b. By linear fitting, the sensitivity can be obtained from the slope of the fitting curve, which is 9.49 RIU^−1^ in the log scale, and the R square can reach 0.9759, showing a satisfactory linear response to *n*_a_ in the range from 1.33 to 1.40.

**Figure 7:**
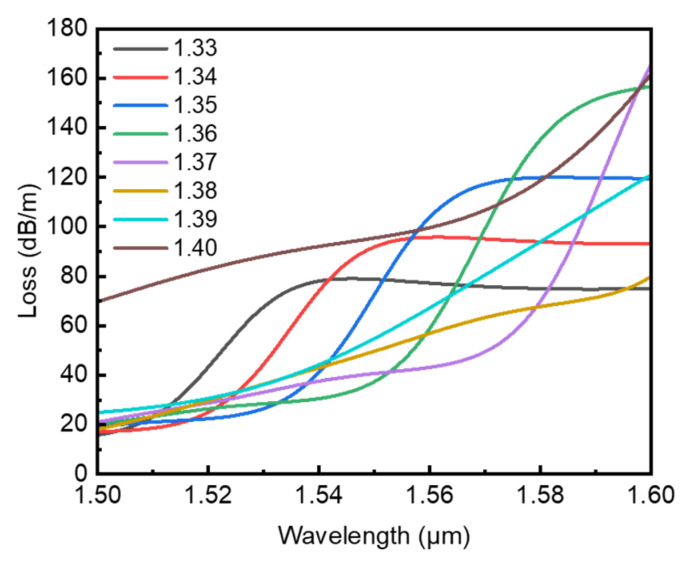
Confinement loss versus wavelength with analyte refractive index changing from 1.33 to 1.40 for twist rate *A* = 12.1 cm^−1^.

For twist rate *A* = 12.1 cm^−1^ where significant couplings occur with *n*_a_ = 1.33 corresponding to the blue curve in Figure 4e, the wavelength-dependent loss spectra near wavelength 1.55 μm are also calculated, as shown in Figure 7. In the vicinity of the resonant coupling conditions, we can see a series of loss peaks appeared in the loss spectra for different *n*_a_. Initially, one can see that the loss peaks increase and shift towards longer wavelengths with rising *n*_a_. However, for a relatively high refractive index *n*_a_, such as *n*_a_ = 1.40, the overall loss is higher than the resonant peaks corresponding to *n*_a_ = 1.33 and 1.34 in the wavelength range shown in Figure 7. This indicates that for increments of the refractive index *n*_a_ taking 0.01, the increase in and movement of the loss peaks are mainly nonlinear and irregular in this case. Hence, we calculate the loss spectra with *n*_a_ changing from 1.33 to1.34 by taking 0.001 as an increment in the refractive index this time.

**Figure 8:**
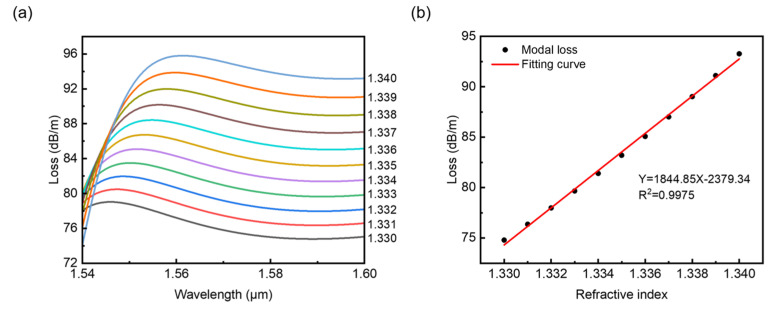
(**a**) Confinement loss versus wavelength with analyte refractive index changing from 1.33 to 1.34 for *A* = 12.1 cm^−1^. (**b**) Linear fitting for selected wavelength λ = 1.589 μm.

As shown in Figure 8a, for a fixed wavelength, the confinement loss increases in a relatively regular magnitude with *n*_a_ changing from 1.330 to 1.340. Figure 8b shows the linear fitting of loss versus refractive index *n*_a_ for selected wavelength λ = 1.589 μm. One can see that the sensitivity corresponding to the slope of the fitting curve can reach a relatively high value of 1844.85 dB/(m·RIU), showing that the core modes are sensitive to small changes in the refractive index of the surroundings, and the obtained R squared value 0.9975 shows that the result has a considerably high linearity.

**Figure 9:**
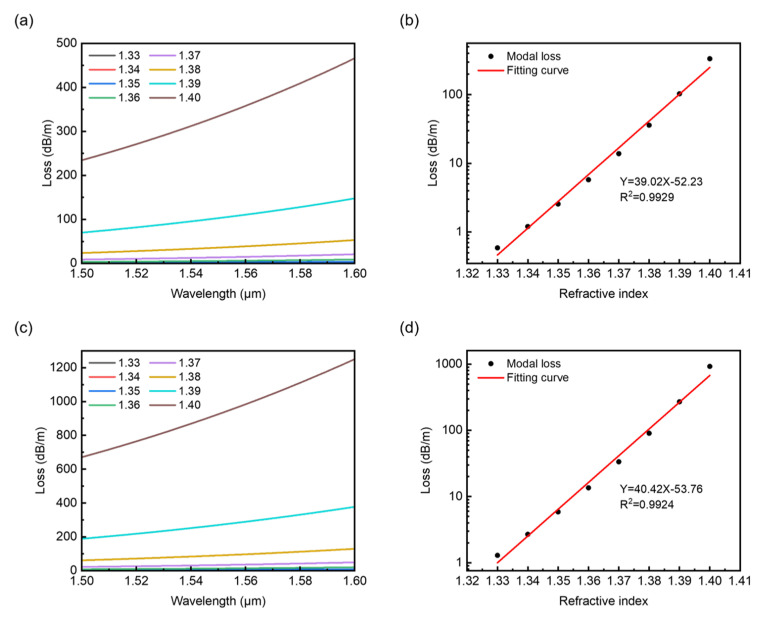
Confinement loss versus wavelength and the linear fitting for selected wavelength λ = 1.55 μm with analyte refractive index changing from 1.33 to 1.40 for *A* = 20 cm^−1^ (**a**,**b**) and 25 cm^−1^ (**c**,**d**).

For the twist rates away from the coupling conditions, the loss spectra and linear fittings are shown in Figure 9. Compared to the results in Figure 6, the confinement losses calculated here for chosen twist rate *A* = 20 and 25 cm^−1^ increase much more rapidly due to the fiber twist. As shown in Figure 9a,c, the curves may look identical to each other, but for a higher twist rate *A*, the overall loss is also higher, which agrees with the blue curve in the twist rate range from 20 to 25 cm^−1^ in Figure 4e. The linear fittings for selected wavelength λ = 1.55 μm are shown in Figure 9b,d. Analogous to the results shown in Figure 6, it is obvious the increases in loss with *n*_a_ are nonlinear, and thus, the fitting of loss versus refractive index of analyte *n*_a_ is presented in the log scale, which seems to show better linearity. For the two chosen twist rates, the increases in loss versus refractive index *n*_a_ show good linearity with R squared values 0.9929 and 0.9924. The obtained sensitivities are 39.02 and 40.42 RIU^−1^ in the log scale, respectively. In this case, one can notice that the increase in twist rate enhances the sensitivities of the fiber. In fact, though the broken air ring plays a role in reducing the confinement loss, the modal fields tend to move outwards with the increasing twist rate. Thus, with a high twist rate and refractive index growing in the broken air ring, more of the mode field will leak into the analyte, resulting in the amplification of confinement loss, improving the response to the changes in the refractive index of the analyte.

**Figure 10:**
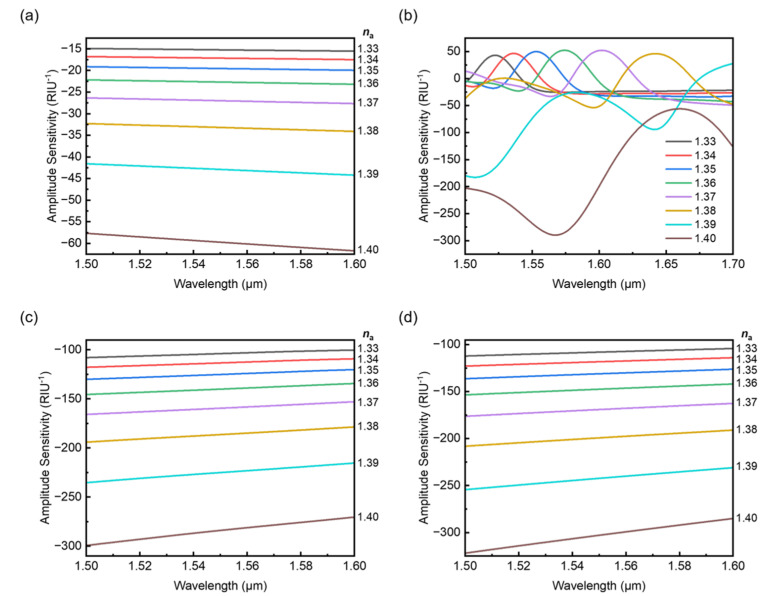
Amplitude sensitivity for selected twist rate *A* = 5 (**a**), 12.1 (**b**), 20 (**c**) and 25 cm^−1^ (**d**).

Figure 10 shows the wavelength-dependent amplitude sensitivities of the proposed fiber with the same twist rates selected in Figure 6 to Figure 9. The amplitude sensitivity can be calculated based on the amplitude interrogation method [25] and it is mostly used for evaluating the sensing performance of plasmonic photonic crystal fibers [26]. Here we also apply the method to the proposed fiber to show the sensing performance accordingly. 

As shown in Figure 10a, the sensitivity increases greatly with the refractive index of analyte *n*_a_ and the changes in the curves are smooth and stable. One can see the maximum sensitivity of 61.72 RIU^−1^ is achieved at 1.6 μm with *n*_a_ = 1.40. In Figure 10b, for twist rate *A* = 12.1 cm^−1^ in the vicinity of the couplings the sensitivities vary dramatically and a maximum value of sensitivity which can be found at 1.567 μm is 289.69 RIU^−1^. For the high twist rates *A* = 20 and 25 cm^−1^ the sensitivities also rise with the refractive index of analyte *n*_a_ as shown in Figure 10c,d. However, one can obtain higher sensitivities in both cases compared to Figure 10a and the maxima of sensitivity can reach 299.3 and 321.99 RIU^−1^, respectively. Moreover, the sensitivities decrease with wavelength in both cases in contrast to the lower twist rate case and this can be attributed to the increase in the overall loss caused by the fiber twist. Furthermore, it is not difficult to find the variation of amplitude sensitivity shown here is also agreed with the analysis for the linear fittings.

## 5. Conclusions

In summary, we study a helical broken-circular-symmetry core microstructured optical fiber using finite element modeling combined with transformation optics formalism. Numerical results show that for the untwisted fiber, modes propagating in such a core formed by a broken air ring suffer from high leakage loss, and for the twisted fiber, modal loss will continue to rise due to the fiber twist. However, if the broken air ring is placed a distance away from the fiber center, one can observe the reduction in confinement loss with increasing twist due to the twist-induced light guidance mechanism. More importantly, we first observe additional couplings between the core modes and the highly leaky modes generated by the helical structure in the loss spectra, where loss peaks appear with relatively high *αρ*. The effect of refractive index changing in the broken air ring on the core modes is also investigated in detail. For a selected set of fiber structural parameters R_2_ = 2R_1_ and *ρ* = 15 μm with a relatively low twist rate *A* = 5 cm^−1^, the confinement loss increases significantly with *n*_a_, and it could be fitted linearly in the log scale, showing a good linear response to the variation of surroundings in the refractive index. For an intermediate twist rate where the resonant couplings take place, the movement of loss spectra is less regular, with an increment of 0.01 in *n*_a_. However, the sensitivity could reach a relatively high value of 1844.85 dB/(m·RIU) for *n*_a_ ranging from 1.33 to 1.34 with an increment of 0.001. For the relatively high twist rate region away from the coupling condition, the considerable sensitivities also could be obtained by linear fitting in log scale, and the scale of loss is smaller compared to the case of lower twist rate (i.e., *A* = 5 cm^−1^). Also, by using the amplitude interrogation method, we present amplitude sensitivity for the selected twist rates and the maximum amplitude sensitivity shown in this paper can reach over 300 RIU^−1^. Moreover, the sensitivity could be further improved by the loss amplification due to the fiber twist. The obtained results show that with such optical properties and compact structure, the fiber has great potential in the field of fiber sensing.

## Data Availability

Data underlying the results presented in this paper are available from the authors upon reasonable request.

## References

[1] Birch R.D. (1987). Fabrication and characterisation of circularly birefringent helical fibres. Electron. Lett..

[2] Kopp Victor I., Churikov Victor M., Singer J., Chao N., Neugroschl D., Genack Azriel Z. (2004). Chiral Fiber Gratings. Science.

[3] Kopp V.I., Churikov V.M., Zhang G., Singer J., Draper C.W., Chao N., Neugroschl D., Genack A.Z. (2007). Single- and double-helix chiral fiber sensors. J. Opt. Soc. Am. B.

[4] Kopp V.I., Park J., Wlodawski M., Singer J., Neugroschl D., Genack A.Z. (2014). Chiral Fibers: Microformed Optical Waveguides for Polarization Control, Sensing, Coupling, Amplification, and Switching. J. Lightwave Technol..

[5] Zhang L., Liu Y., Cao X., Wang T. (2016). High Sensitivity Chiral Long-Period Grating Sensors Written in the Twisted Fiber. IEEE Sens. J..

[6] Napiorkowski M., Urbanczyk W. (2016). Surface plasmon resonance effect in helical core fibers. J. Opt. A Pure Appl. Opt..

[7] Shin W., Lee Y.L., Yu B.-A., Noh Y.-C., Oh K. (2009). Spectral characterization of helicoidal long-period fiber gratings in photonic crystal fibers. Opt. Commun..

[8] Wong G.K., Kang M.S., Lee H.W., Biancalana F., Conti C., Weiss T., Russell P.S. (2012). Excitation of orbital angular momentum resonances in helically twisted photonic crystal fiber. Science.

[9] Xi X., Wong G.K.L., Weiss T., Russell P.S.J. (2013). Measuring mechanical strain and twist using helical photonic crystal fiber. Opt. Lett..

[10] Xi X.M., Wong G.K.L., Frosz M.H., Babic F., Ahmed G., Jiang X., Euser T.G., Russell P.S.J. (2014). Orbital-angular-momentum-preserving helical Bloch modes in twisted photonic crystal fiber. Optica.

[11] Beravat R., Wong G.K.L., Xi X.M., Frosz M.H., St.J. Russell P. (2016). Current sensing using circularly birefringent twisted solid-core photonic crystal fiber. Opt. Lett..

[12] Beravat R., Wong G.K.L., Frosz M.H., Xi X.M., Russell P.S.J. (2016). Twist-induced guidance in coreless photonic crystal fiber: A helical channel for light. Sci. Adv..

[13] Napiorkowski M., Zolnacz K., Statkiewicz-Barabach G., Bernas M., Kiczor A., Mergo P., Urbanczyk W. (2020). Twist Induced Mode Confinement in Partially Open Ring of Holes. J. Lightwave Technol..

[14] Fujisawa T., Saitoh K. (2018). Off-axis core transmission characteristics of helically twisted photonic crystal fibers. Opt. Lett..

[15] Davtyan S., Chen Y., Frosz M.H., Russell P.S.J., Novoa D. (2020). Robust excitation and Raman conversion of guided vortices in a chiral gas-filled photonic crystal fiber. Opt. Lett..

[16] Fu C., Liu S., Wang Y., Bai Z., He J., Liao C., Zhang Y., Zhang F., Yu B., Gao S. (2018). High-order orbital angular momentum mode generator based on twisted photonic crystal fiber. Opt. Lett..

[17] Zeng X., He W., Frosz M.H., Geilen A., Roth P., Wong G.K.L., Russell P.S.J., Stiller B. (2022). Stimulated Brillouin scattering in chiral photonic crystal fiber. Photonics Res..

[18] Zeng X., Russell P.S.J., Wolff C., Frosz M.H., Wong G.K.L., Stiller B. (2022). Nonreciprocal vortex isolator via topology-selective stimulated Brillouin scattering. Sci. Adv..

[19] Zolnacz K., Napiorkowski M., Kiczor A., Makara M., Mergo P., Urbanczyk W. (2020). Bend-induced long period grating in a helical core fiber. Opt. Lett..

[20] Nicolet A., Zolla F., Agha Y.O., Guenneau S. (2008). Geometrical transformations and equivalent materials in computational electromagnetism. COMPEL.

[21] Nicolet A., Zolla F., Agha Y.O., Guenneau S. (2007). Leaky modes in twisted microstructured optical fibers. Waves Random Complex Medium.

[22] Napiorkowski M., Urbanczyk W. (2014). Rigorous simulations of a helical core fiber by the use of transformation optics formalism. Opt. Express.

[23] Napiorkowski M., Urbanczyk W. (2018). Scaling effects in resonant coupling phenomena between fundamental and cladding modes in twisted microstructured optical fibers. Opt. Express.

[24] Napiorkowski M., Urbanczyk W. (2016). Coupling between core and cladding modes in a helical core fiber with large core offset. J. Opt. A Pure Appl. Opt..

[25] Hassani A., Skorobogatiy M. (2006). Design of the Microstructured Optical Fiber-based Surface Plasmon Resonance sensors with enhanced microfluidics. Opt. Express.

[26] Hu D.J.J., Ho H.P. (2017). Recent advances in plasmonic photonic crystal fibers: Design, fabrication and applications. Adv. Opt. Photonics.

